# Are Translation Equivalents Always Activated When Bilinguals Perform a Task in One of Their Languages? Behavioral and ERP Evidence of the Role of the Task

**DOI:** 10.3390/brainsci13030432

**Published:** 2023-03-02

**Authors:** Pilar Ferré, Josep Albert Obrador, Josep Demestre

**Affiliations:** Department of Psychology and CRAMC, Universitat Rovira i Virgili, 43007 Tarragona, Spain

**Keywords:** Revised Hierarchical Model, highly proficient bilinguals, translation recognition task, primed lexical decision task, semantic relationship, form relationship, conceptual route, lexical route

## Abstract

This study investigates the extent to which highly proficient Spanish–Catalan bilinguals activate Spanish translation equivalents when they are presented with Catalan words. Participants performed a translation recognition task (Experiment 1) or a primed lexical decision task (Experiment 2) where the relationship between the first presented (Catalan) word and the second presented (Spanish) word was manipulated. Semantic and form relationships between the first and the second words were examined. Semantic relatedness produced a behavioral interference effect in the translation recognition task and a facilitation effect in the primed lexical decision task. The semantic manipulation also affected the N400 component. Form relatedness produced a behavioral interference effect only in the translation recognition task, which was accompanied by a modulation of the LPC component. In contrast, there were no effects of the formal manipulation in the primed lexical decision task. These results, which are discussed in relation to the revised hierarchical model (RHM), suggest that activation of translation equivalents is a by-product of the type of task.

## 1. Introduction

In recent years, the organization and access to bilingual memory has been a main topic of interest in psycholinguistics. A leading theory in the field, i.e., the revised hierarchical model (RHM, [1]) tries to explain how the lexical forms of words in the two languages are mapped to meaning. According to the RHM, there are two separated (but interconnected) lexicons, one for the first language (L1), and another for the second language (L2). The lexicons are both connected, in turn, to a shared conceptual system. The model assumes the existence of two types of connections: lexical connections (i.e., between the L1 and L2 lexical systems) and conceptual connections (between each of the two lexical systems and the conceptual system). One of the main attractions of this model relies on its developmental nature. The RHM posits that the strength of such connections and, consequently, the processing of words in the L2, vary as a function of proficiency. At the initial stages of L2 acquisition, the connections between the L2 and the conceptual system are very weak. For that reason, L2 words access their meaning by an indirect route, that is, when an L2 word is presented, it activates its translation equivalent in L1 through the lexical connection, which in turn activates the semantic representation. As proficiency in the L2 increases, the direct links between the L2 lexical forms and the shared conceptual system enhance their strength. Hence, dependency on the lexical route to access concepts from the L2 decreases in proficient bilinguals, who can directly access the conceptual system from their L2.

The RHM has inspired a great deal of research and much evidence in its favor has accumulated (see [2] for an overview). However, it has increasingly been challenged by experimental results that do not fit into the model’s predictions, leading some authors to suggest that it should be left behind in favor of other computational models [2]. Focusing on the developmental aspect of the RHM, the main challenge has to do with access to the conceptual system when processing L2 words, which takes place mostly via the conceptual link in proficient bilinguals and via the lexical link (i.e., mediated by the activation of the L1 translation equivalent) in non-proficient bilinguals. Several findings question this assumption. On the one hand, some studies have suggested that even in bilinguals with low proficiency levels, there is evidence of direct access to the conceptual system from the L2 [3,4,5,6]. On the other hand, it seems that proficient bilinguals are also sensitive to manipulations that indicate the activation of L1 translation equivalents [7,8,9,10,11]. The present study focuses on this last issue, that is, on the extent to which highly proficient bilinguals activate the translation equivalents in the other language when they process words in one of their languages. To that end, bilinguals of Catalan and Spanish performed a translation recognition task (Experiment 1) and a lexical decision task with a priming paradigm (Experiment 2), and both behavioral and electrophysiological measures (event-related potentials, ERPs) were recorded.

In the translation recognition task, participants are presented with pairs of words, one in the L2 and the other in the L1 (e.g., *ruc–burro* in Catalan and Spanish, donkey), and they are asked to decide whether or not the two words are translation equivalents. In a particular version of this task (the one that has been mostly used to test the RHM in the literature), the critical stimuli are the non-translation pairs (i.e., those where the correct response is “no”). Here, participants are presented with words that can be related across languages either in meaning (e.g., *ruc–caballo*, donkey–horse) or in form (*ruc–berro*, donkey–watercress, where *berro* is a Spanish word similar in form to the Spanish translation of *ruc*, that is, *burro*). The common result is that participants take longer (and commit more errors) to decide that the meaning-related L1 words and the form-related L1 words are not the correct translation of the L2 words than unrelated (control) L1 words (e.g., *ruc–lejía,* bleach). Therefore, two types of interference effects can be observed in this task, a semantic interference effect (resulting from the comparison of semantically related pairs to unrelated pairs) and a form interference effect (resulting from the comparison of form-related pairs to unrelated pairs). The former effect is considered to be evidence that bilinguals are directly accessing the meaning from L2 words (i.e., they are using the conceptual route, which is sensitive to semantic manipulations), while the latter effect is considered to be evidence that bilinguals are using the lexical route (which is sensitive to form-related manipulations) to access meaning from L2 words [4,7,8,9,10,11,12].

Several studies have been conducted with the translation recognition task in proficient bilinguals and they have revealed that their performance was affected by both semantic and form-related distractors [7,8,9,10,11,12]. The RHM does not necessarily predict a total lack of form interference effect in these bilinguals. Rather, what it predicts is a preponderance of the semantic route over the lexical route. There are contrasting findings here because, whereas some studies have found a larger semantic than form interference effect, as predicted by the model [12], others have found interference effects of the same magnitude for both types of distractors [7,8,9,10,11].

A form-related interference effect of the same magnitude as a semantic interference effect suggests that the L1 translation equivalents have been activated (i.e., if *berro* produces an interference effect, it is because *burro*, the translation of *ruc*, has been activated). Importantly, the activation of the L1 translation equivalents itself does not necessarily challenge the RHM. What is crucial for the tenets of the model is the extent to which such activation is needed for proficient bilinguals to access meaning from their L2. To answer this question, behavioral measures may not be sensitive enough, since they reflect only final reaction times. In contrast, event-related potentials (ERPs) give information of the time course of the cognitive processes involved in language processing [13].

A study by [9] used ERPs to elucidate whether the activation of translation equivalents was required to access meaning from the L2 in relatively proficient Chinese-English bilinguals. These authors examined form and semantic relationships in two translation recognition experiments involving different stimulus-onset asynchronies (SOA). They obtained a semantic and a form-related interference effect with both a long (750 ms) and a short (300 ms) SOA. Crucially, the ERP revealed a different time course for these two effects. The semantic manipulation produced a clear effect in the N400 component, which indexes semantic integration. The form manipulation, by contrast, did not modulate the N400, but a late positive component (LPC, in a time window from 500 to 700 ms). Considering that the form manipulation affected a component that appeared later than the N400, the authors concluded that proficient bilinguals activated the translation equivalents after meaning retrieval.

Similar conclusions were reached by a study conducted a few years later by [11]. The main difference with respect to [9] is that, in [11], participants were highly proficient bilinguals, who were very balanced and immersed in a bilingual environment where both languages, i.e., Catalan and Spanish, were present. Similar to [9], the semantic manipulation, but not the form manipulation, modulated the N400 component. The form manipulation, in turn, modulated the LPC component. The results of [9,11] suggest that proficient bilinguals access meaning directly when they perform a translation recognition task. It is only when the task provides enough time that the translation equivalents are also activated, after meaning has been retrieved [9]. Therefore, form-related interference effects found in previous studies [7,8,9,10,11,12] do not challenge the tenets of the RHM, because ERP data indicate that the activation of translation equivalents is not required to access meaning.

Another factor that has not been taken into account in this line of research may contribute to the form-related effects in proficient bilinguals, concretely, the characteristics of the task. All the studies that have relied on form and semantic interference effects as indices of the activation of semantic and lexical links have used a translation recognition task. As explained above, in this task, participants are presented consecutively with pairs of words belonging to their two languages and are asked to decide if the second word is the correct translation of the first one. Therefore, they need to compare both words (i.e., the correct translation of the word presented in the first place with the word presented in second place) to decide if there is a match. It is not surprising that the L1 translation equivalent is activated in such conditions. This activation might not be observed in tasks that do not involve such a comparison and do not focus on translation.

### The Present Study

The aim of the present study was to examine the activation of translation equivalentsin highly proficient bilinguals. Previous studies in the field have relied on the translation recognition task. This study is the first time another task, not focused on translation, is included with comparative purposes. This allows us to examine if previous results may be explained by the type of task used. We tested a group of highly proficient bilinguals of Spanish and Catalan, obtained from the same population as participants in the studies of [7,10,11,14]. We tested them in a translation recognition task (the one used in all the previous studies, Experiment 1) and in a lexical decision task with a priming paradigm (Experiment 2). This last task was used because it has been shown to be sensitive to both semantic effects [14,15,16,17,18] and form effects [19,20,21,22,23] produced by the presentation of a word in one language (prime) in the processing of a word in another language (target). We recorded both behavioral and ERP measures.

The critical experimental materials were the same in the two tasks. Participants were presented with pairs of Catalan–Spanish words. The two languages involved in the study were not labeled as L1 or L2, considering the high level of proficiency of participants in both languages. The critical pairs could be either related in form or in meaning across languages, or totally unrelated. The second (Spanish) word remained constant, and the first (Catalan) word varied across conditions. This was a change with respect to the materials used in previous translation recognition studies [7,8,9,10,11], where the first word was always the same and the second word changed across conditions. This variation was necessary to have exactly the same materials in the translation recognition task as in the primed lexical decision task. In the translation recognition task, participants were asked to decide whether or not the second word was the correct translation of the first word. In the lexical decision task, participants were asked to decide whether or not the second string of letters was an existing Spanish word.

The following research questions guided the study:

RQ1: Does semantic relatedness affect performance of highly proficient bilinguals, regardless of the type of task? To answer this research question we presented bilinguals of Catalan and Spanish with pairs of Catalan–Spanish words. We compared reaction times, percentage of errors, and ERP amplitudes between pairs in which the Spanish translation of the Catalan word was semantically related to the Spanish word and pairs in which both words were unrelated. Participants did a translation recognition task in Experiment 1 and a primed lexical decision task in Experiment 2.

Hypothesis for RQ1: We expected a behavioral effect in both tasks. Considering past findings, this would be an interference effect in the translation recognition task. That is, we expected larger RTs and lower accuracy in the semantically related condition (e.g., *cavall–burro*, horse–donkey) compared to the unrelated condition (e.g., *costum–burro*, custom–donkey [7,8,9,10,11]). In contrast, we expected a facilitation effect in the primed lexical decision task (i.e., shorter RTs and higher accuracy in the semantically related condition compared to the unrelated condition [14,15,16,17,18]). At the electrophysiological level, an effect in the N400 component was expected (i.e., a smaller amplitude in the semantically related condition compared to the unrelated condition, indicating an easier semantic integration [9,11].

RQ2: Does form relatedness affect performance of highly proficient bilinguals and, if this is the case, is the effect modulated by the type of task? To answer this research question, we presented bilinguals of Catalan and Spanish with pairs of Catalan–Spanish words. We compared reaction times, percentage of errors, and ERP amplitudes between pairs in which the Spanish translation of the Catalan word was similar in form to the Spanish word and pairs in which both words were dissimilar. Participants did a translation recognition task in Experiment 1 and a primed lexical decision task in Experiment 2.

Hypothesis for RQ2: Two alternative predictions were made: (1) If the translation equivalent is always activated when highly proficient bilinguals perform any task in any of their languages, the presentation of a Catalan word in the first place (e.g., *fang,* mud), whose Spanish translation equivalent (*barro*) is similar in form to the Spanish word to be presented in second place (*burro*, donkey), will affect the processing of the latter. Regarding the translation recognition task, a behavioral interference effect [7,8,9,10,11] and a modulation of the LPC component should be observed [9,11]. With respect to the primed lexical decision task, a facilitative behavioral effect might be expected (i.e., the activation of *barro* would facilitate the lexical decision about *burro*), as well as a modulation of the LPC component. Alternatively, (2) if the activation of the Spanish translation equivalent is a by-product of the characteristics of the task (i.e., a task focused on translation, where both words are compared), the effects of the form manipulation should be restricted to the translation recognition task, while there would not be any behavioral or electrophysiological effect in the primed lexical decision task.

## 2. Experiment 1. Translation Recognition Task

### 2.1. Materials and Methods

#### 2.1.1. Participants

Twenty-six participants (23 females, mean age = 22.5, SD = 4.98) took part in the experiment. They were undergraduate students at the Universitat Rovira i Virgili (Tarragona, Spain) who were paid to participate in the experiment. All of the participants signed an informed consent form before starting the experiment, and all participants had normal or corrected-to-normal vision and were right-handed. All participants were bilinguals of Catalan and Spanish. To assess their proficiency in both languages, they were asked to complete a questionnaire in which they rated their ability in reading, writing, speaking, and listening on a 7-point Likert scale (1 = ”very poor”; 7 = ”native-like”). They were also asked for the age of exposure to each language, as well as for language preference and use. Concretely, they assessed their preference using a scale from 1 (exclusively in Catalan) to 7 (exclusively in Spanish), where the middle point (i.e., 4) indicated no preference at all for any of the two languages and the same frequency of use for both languages. The results of the questionnaire (see Table 1) revealed that all the participants were exposed to Catalan from birth (M = 0.53, SD = 0.98) and their average age of initial exposition to Spanish was 1.11 years (SD = 1.98). Ability ratings showed that they were all highly proficient in both languages (see Table 1). A paired comparison between Catalan (M = 6.73, SD = 0.58) and Spanish (M = 6.74, SD = 0.57) showed no differences between Catalan and Spanish in average reported proficiency, t (25) = −0.061, *p* > 0.900. The questionnaire also showed that both Catalan and Spanish were equally preferred (M = 3.98, SD = 1.25) and that participants used the two languages with the same frequency (M = 3.97, SD = 1.24). Thus, participants were early balanced bilinguals of Catalan and Spanish, being highly proficient in both languages with no preference for one of the two languages.

#### 2.1.2. Materials

In this experiment, 760 word pairs were constructed in which the first item in each pair was a Catalan word and the second item was a Spanish word. All the words used in the study were nouns or adjectives. One hundred and sixty of the pairs were correct translation pairs (YES trials), while the remaining 640 pairs, the critical ones, were incorrect translations (NO trials). Among the NO trials, there were two types of critical conditions: the semantically related condition in which the Catalan and the Spanish words were semantically related (e.g., *cavall–burro*, where *cavall* is the Catalan word for *horse*, whereas *burro* is the Spanish word for *donkey*) and the form-related condition in which the Spanish word resembled the Spanish translation of the Catalan word in orthography but not in meaning (e.g., *fang–burro*, *burro* and *barro*, the Spanish translation for *fang* (*fang* is the Catalan word for *mud)* are similar in form).

For the semantically related condition, some of the word pairs were taken from the database of [24]. This database provides measures of semantic similarity for a set of Spanish pairs of words, obtained through a semantic similarity rating task. These pairs are non-associatively related. In order to obtain the final set of stimuli, 290 new word pairs were distributed in five questionnaires with 58 word pairs each. We then asked 100 native Spanish speakers (20 participants per questionnaire) to rate the meaning similarity between the two Spanish words of each pair. We used the same procedure as [24] and asked participants to make their ratings using a Likert-like scale ranging from 1 (nothing similar) to 9 (exactly the same thing). The final set of stimuli in the semantically related condition were the 160 pairs that had the highest ratings of semantic similarity (M = 6.63, SD = 0.76, range = 8.45–5.35). Then, the first word of each pair was translated into Catalan, to obtain the Catalan–Spanish words to be used in the experiment. Importantly, the Catalan–Spanish words in the semantically related condition were not similar in form (as will be explained below).

For the form-related condition, we selected Catalan words whose translation into Spanish was orthographically similar (and semantically unrelated) to the Spanish target. For example, the Spanish target “*burro*” was paired with the Catalan word “*fang*”, whose translation into Spanish (“*barro*”) is orthog raphically similar to the Spanish target word. The orthographic similarity between the Spanish target word (“*burro*”) and the correct translation into Spanish (“*barro*”) of the Catalan word (“*fang*”) was obtained from NIM [25], through the use of the normalized Levenshtein distance (NLD). The Levenshtein distance (LD, [26]) is a measure of the minimum number of operations required to change a string of letters into another one. The normalized LD (NLD; [27]) is obtained by dividing the LD of two letter strings by the number of letters in the longest string and subtracting the result from one. The NLD is easily interpretable since it varies on a continuum between 0 (no similarity) and 1 (exact match). The average NLD between the Spanish target words (e.g., *burro*) and the correct translation into Spanish (*barro* in the example) of the Catalan words (*fang* in the example) was 0.69 (SD = 0.09), indicating that they were orthographically similar. The NLD between the Catalan words (*fang* in the example) and the Spanish target words (*burro* in the example) was 0.16 (SD = 0.1), indicating that they were not orthographically related. The NLD between the Catalan words (*fang* in the example) and their Spanish translations (*barro*) was 0.22 (SD = 0.14), showing that they were not orthographically related either. We also computed the NLD between the Catalan words in the semantically related condition (*cavall* in the example) and their corresponding target Spanish words (*burro*). The result indicated that both words were not orthographically related (M = 0.14, SD = 0.12).

Finally, two matched control conditions were also created (i.e., semantically unrelated and form unrelated). In these two control conditions, the Spanish word from the critical conditions was paired with Catalan words that were neither semantically nor formally related to it (e.g., the Spanish word *burro* was paired with the Catalan words *costum* and *roda* (meaning *custom and wheel,* respectively)), creating a matched unrelated condition for each related condition. Word length (i.e., number of characters) and frequency were not significantly different between the related and unrelated words in the semantic condition, or between the related and unrelated words in the form condition (all ts < 1). Table 2 provides the lexical properties of the stimuli in the four critical conditions. The same Spanish word was used in the four conditions as the target item (i.e., the word to which participants responded). There were 160 word pairs in each condition.

Four lists were constructed, with 40 critical word pairs in each critical condition. The four types of NO trials were counterbalanced such that each Spanish target word was presented only once in a list, paired with either its semantically related condition, the form-related condition, the semantically unrelated condition, or the form-unrelated condition. The four lists also contained the same 160 correct translation (non-critical) pairs. In this way, participants were presented with the same number (i.e., 160) of YES and NO trials.

#### 2.1.3. Procedure

Participants were tested in a sound-attenuated and electrically shielded room. Then, they performed a translation recognition task, which was identical to that in [11]. Participants were presented with pairs of Catalan–Spanish words and were asked to decide whether the second word of a pair was the correct translation of the first word of the pair. They had to answer by pressing, with the right hand, one button of the keyboard labeled YES for correct translations and by pressing, with the left hand, another button labeled NO for incorrect translations as quickly and accurately as possible. The EEG was monitored while participants performed the task. The stimuli were presented one at a time at the center of the screen, in white font, on a black background, using the DMDX program [29]. The computer generated a pseudo-random order of presentation for each participant. Each trial started with an image of an eye displayed for 2000 ms, which indicated to participants that they were allowed to blink their eyes, followed by a 500 ms fixation point (“#”). They were also instructed to restrain their blinks to when the image of an eye was presented. Just after the fixation point, a Catalan word was presented for 250 ms, immediately followed by the presentation of a Spanish word. The Spanish word remained on the screen until the participant responded or 2000 ms had elapsed. There was a 1000 ms ISI between the trials. The experiment began with 12 practice trials (6 YES trials and 6 NO trials). There was a total number of 320 experimental pairs. There were brief breaks after every 80 pairs. Participants were evenly distributed across the four counterbalanced lists.

#### 2.1.4. EEG Recording and Analysis

Each participant was seated in a comfortable chair about 60 cm from the stimulus monitor. The electroencephalogram was recorded from 30 Ag/AgCl active electrodes attached on the ActiCap system (Brain Products GmbH, Gilching, Germany) placed on the scalp according to the extended 10–20 system. Vertical and horizontal electrooculograms (EOGs) were recorded through two additional electrodes attached below the left eye and the corner of the right eye. All electrodes were referenced online to the left mastoid. An additional electrode was attached to the right mastoid for offline referencing. Electrode impedances were kept below 5 kΩ. The EEG and EOG signals were recorded and digitized with PyCorder and amplified using an antiCHamp amplifier (Brain Vision LLC). All channels were amplified with a band pass of 0.01–100 Hz and at a sampling rate of 500 Hz.

Offline, ERPs were time-locked to the second word of each pair and averaged over a 900 ms epoch, including a 100 ms prestimulus-onset baseline. The EEG was refiltered offline with a 25 Hz, low-pass, zerophase shift digital filter. Automatic and manual rejections were carried out to exclude periods containing movement or technical artifacts (the automatic EOG rejection criterion was ±70 μV). After these rejection procedures, additional visual inspection of the resulting signal was carried out for each participant individually. Only trials without muscle artifact or eye movement/blink activity were included in the averaging process. After artifact rejection, the percentage of trials retained for analyses was 92.14, with 91.44% for the semantically related condition, 92.60% for the semantic control condition, 92.21% for the form-related condition, and 92.31% for the form control condition. There were no significant effects of type of relationship or relatedness, nor were there any interaction effects between these factors (all *p*-values > 0.48) on the percentage of trials retained for analysis.

Nine regions, each containing the average values of a group of three electrodes, were computed by collapsing across the following electrodes: left frontal (LF: average among F7, F3, and FC5); midline frontal (MF: Fp1, Fp2, and Fz); right frontal (RF: F4, F8, and FC6); left central (LC: T7, C3, and CP5); midline central (MC: FC1, FC2, and Cz); right central (RC: C4, T8, and CP6); left posterior (LP: P7, P3, and O1); midline posterior (MP: CP1, CP2, and Pz); right posterior (RP: P4, P8, and O2). The analyses involved repeated measures ANOVAs with within-participants factors relatedness (related vs. unrelated), anteriority (anterior, central, and posterior), and hemisphere (left, central, and right). The ERP effects were statistically analyzed in two time windows (N400 and LPC), determined a priori by previous literature. The N400 was analyzed from 300 ms to 500 ms, and the LPC was evaluated from 500 ms to 700 ms. The Greenhouse and Geisser [30] correction was applied to all repeated measures having more than one degree of freedom in the numerator. In such cases, the corrected *p*-value is reported. Bonferroni adjustment was used for post hoc pairwise comparisons.

### 2.2. Results

#### 2.2.1. Behavioral Results

Seven word pairs were rejected because they had a 100% error rate. RTs shorter than 200 ms and longer than 2000 ms were also rejected. Further, scores that were 2.5 standard deviations above or below the mean value of each participant and each item were excluded. Only correct responses were included in the RT analyses. Mean reaction times (RTs) and error rates across the four critical conditions are shown in Table 3.

Separate 2 (type of relationship, semantic vs. form) * 2 (relatedness, related vs. unrelated) by-participant (F1) and by-item (F2) ANOVAs were performed for RTs and error rates. The factors were both treated as within-group factors in the by-participant analysis as well as in the by-item analysis.

##### Reaction Times

The results showed a significant effect of the type of relationship (F_1_(1, 25) = 13.286, *p* = 0.001, η_p_^2^ = 0.347, F_2_(1, 152) = 17.214, *p* < 0.001, η_p_^2^ = 0.102), indicating that responses to the target word in the semantic condition (M = 718 ms, SE = 27.54) were slower than those in the form condition (M = 682 ms, SE = 22.06). The main effect of relatedness was also significant (F_1_(1, 25) = 119.723, *p* < 0.001, η_p_^2^ = 0.827, F_2_(1, 152) = 77.319, *p* < 0.001, η_p_^2^ = 0.337) suggesting that it took participants longer to reject related words (M = 739 ms, SE = 26.16) than unrelated words (M = 661 ms, SE = 23.18). The interaction effect between the type of relationship and relatedness was also significant (F_1_(1, 25) = 4.720, *p* = 0.040, η_p_^2^ = 0.159, F_2_(1, 152) = 4.527, *p* = 0.035, η_p_^2^ = 0.029). Pairwise Bonferroni-corrected comparisons showed that the magnitude of semantic interference (90 ms, *p* < 0.001) was larger than that of the form interference (65 ms, *p* < 0.001).

##### Error Rates

The results showed a significant effect of the type of relationship (F_1_(1, 25) = 115.965, *p* < 0.001, η_p_^2^ = 0.823, F_2_(1, 152) = 61.731, *p* < 0.001, η_p_^2^ = 0.289), indicating that error rates for word pairs in the semantic condition (M = 13.41%, SE = 1.02) were higher than those in the form condition (M = 3.51%, SE = 0.54). The main effect of relatedness was also significant (F_1_(1, 25) = 143.924, *p* < 0.001, η_p_^2^ = 0.852, F_2_(1, 152) = 173.647, *p* < 0.001, η_p_^2^ = 0.533) suggesting that participants committed more errors in the related condition (M = 16.06%, SE = 1.29) than in the unrelated condition (M = 0.86%, SE = 0.24). The interaction effect between the type of relationship and relatedness was also significant (F_1_(1, 25) = 96.049, *p* < 0.001, η_p_^2^ = 0.793, F_2_(1, 152) = 53.261, *p* < 0.001, η_p_^2^ = 0.259). Pairwise Bonferroni-corrected comparisons showed that the difference between the related and the unrelated words in the semantic condition (24.17, *p* < 0.001) was larger than in the form condition (6.23, *p* < 0.001).

#### 2.2.2. ERP Results

We conducted a four-way ANOVA with relatedness (related vs. unrelated), type of relationship (semantic vs form), hemisphere (left vs. midline vs. right), and latitude (frontal, central and parietal) as within-participant factors. Only the main effect of the relatedness factor and significant interaction effects between this factor and the other three factors (i.e., type of relationship, hemisphere, and latitude) are reported. The main effect of type of relationship and its interaction with the two topographical factors, as well as the main effects of or interactions exclusively between topographical factors are not of primary relevance to the current work and, thus, are not reported.

**Figure 1:**
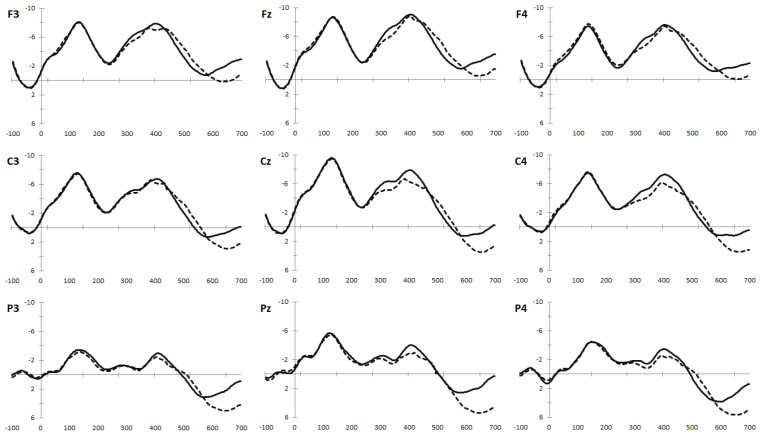
Grand average ERP waveforms for form-unrelated (dotted lines) and form-related words (solid lines) in Experiment 1. ERPs are plotted for nine equidistant representative electrodes within each region of interest. Negative voltage is plotted up. Onset of the Spanish word (i.e., the target) is indicated by the vertical calibration bar. Data are plotted from 100 ms prior to and 700 post stimulus onset.

**Figure 2:**
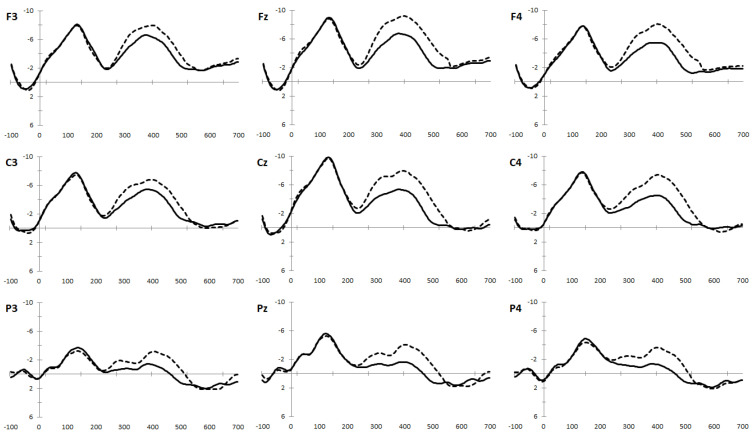
Grand average ERP waveforms for semantically unrelated (dotted lines) and semantically related words (solid lines) in Experiment 1. ERPs are plotted for nine equidistant representative electrodes within each region of interest. Negative voltage is plotted up. Onset of the Spanish word (i.e., the target) is indicated by the vertical calibration bar. Data are plotted from 100 ms prior to and 700 post stimulus onset.

**Figure 3:**
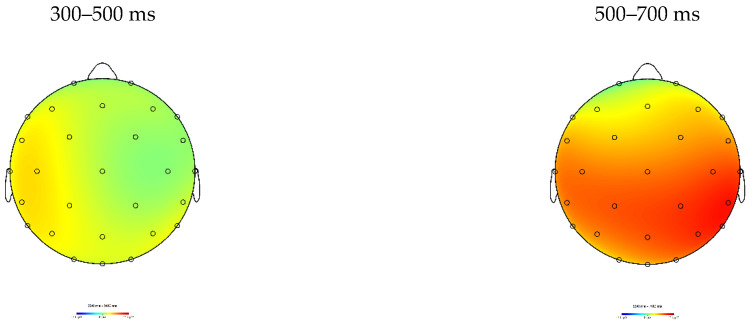

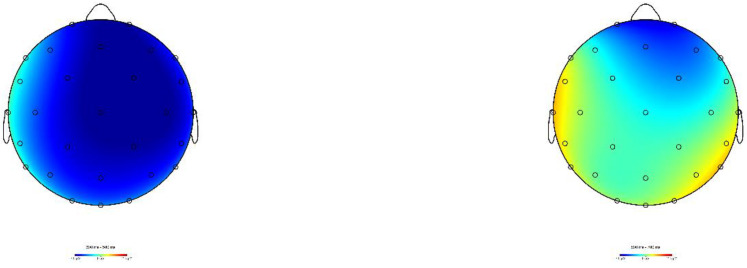
Scalp maps of relatedness effects (voltage differences were computed by subtracting the related from the unrelated condition) averaged across the N400 and the LPC time windows in Experiment 1 (upper row, the form condition and lower row, the semantic condition). Scale from −2.5 μV (blue) to 2.5 μV (red).

Grand-average ERP waveforms in a selection of electrodes are shown for the form condition (Figure 1) and the semantic condition (Figure 2). Figure 3 shows the scalp topographies of the mean amplitudes in the time windows for the N400 and the LPC components for the form condition (upper row) and the semantic condition (lower row).

Figure 1 and Figure 2 reveal clear differences between the ERPs to targets preceded by unrelated versus related words in the two conditions. Visual inspection of the ERP waveforms for the form condition (Figure 1) reveals that unrelated words elicit a late positivity starting at around 500 ms post stimulus. Visual inspection of the ERP waveforms for the semantic condition (Figure 2) reveals that, relative to the related condition, the unrelated condition exhibits a clear modulation of the N400 component, starting at around 300 ms post stimulus. The topographic plots in Figure 3 show that there are no differences at the 300–500 ms time window between unrelated and related words in the form condition, whereas, for the semantic condition, unrelated words elicit a widely distributed negativity. Moreover, Figure 3 shows that, whereas unrelated words in the form condition elicit a more positive waveform at the 500–700 ms time window, in the semantic condition, unrelated words continue to elicit the negativity already observed in the N400 time window, although less pronounced.

##### N400

The ANOVA revealed a significant effect of relatedness (F (1, 25) = 6.686, *p* = 0.016, η_p_^2^ = 0.211). Unrelated words elicited a more negative-going wave (M = −3929 μV, SE = 0.46) than related words (M = −3250 μV, SE = 0.46). The interaction effect between relatedness and type of relationship was significant (F (1, 25) = 32.957, *p* < 0.001, η_p_^2^ = 0.569). Post hoc pairwise Bonferroni-corrected comparisons showed that, whereas the difference between related and unrelated words was significant in the semantic condition (*p* < 0.001), the two levels of the relatedness factor did not differ in the form condition (*p* > 0.320). The ANOVA revealed that the interaction effect between relatedness and hemisphere was also significant (F (2, 50) = 12.906, *p* < 0.001, η_p_^2^ = 0.340). Pairwise Bonferroni-corrected comparisons showed that there were no differences between unrelated and related words at the left hemisphere (*p* = 0.455), whereas the differences were significant at the midline (*p* < 0.004) and at the right hemisphere (*p* < 0.004), with unrelated words eliciting a more negative-going wave. Furthermore, the three-way interaction effect among relatedness, hemisphere, and latitude was significant (F (4, 100) = 3.187, *p* = 0.038, η_p_^2^ = 0.113). Post hoc pairwise Bonferroni-corrected comparisons revealed that the ERP response elicited by unrelated words did not differ from the ERP response elicited by related words at either of the three latitudes of the left hemisphere (all *p*s > 0.270). In contrast, unrelated words elicited a significantly more negative-going wave than related words at the three latitudes of both the midline (all *p*-values < 0.015) and the right hemisphere (all *p*-values < 0.019). Finally, the interaction effect among relatedness, type of relationship, and hemisphere was also significant (F (4, 100) = 11.446, *p* < 0.001, η_p_^2^ = 0.314). Post hoc Bonferroni-corrected comparisons revealed that the differences between unrelated and related words in the semantic condition were significant at the left (*p* = 0.008) and right hemisphere (*p* < 0.001), as well as at the midline (*p* < 0.001). In contrast, there were no significant differences between unrelated and related words in the form condition at either of the three levels of the hemisphere factor (all *p*-values > 0.132). All other interaction effects were not significant (all *p*-values > 0.365).

##### LPC

The overall ANOVA revealed a significant effect of relatedness, F (1, 25) = 5.250, *p* = 0.031, η_p_^2^ = 0.174. Unrelated words elicited a more positive-going wave than related words. The interaction effect between relatedness and type of relationship was significant, F (1, 25) = 24.612, *p* < 0.001, η_p_^2^ = 0.496. Post hoc pairwise Bonferroni-corrected comparisons showed that whereas there were no significant differences between related and unrelated words in the semantic condition (*p* = 0.626), unrelated words elicited a significantly more positive-going wave than related words in the form condition (*p* < 0.001). The interaction effect between relatedness and hemisphere was also significant (F (2, 50) = 4.373, *p* = 0.018, η_p_^2^ = 0.149). Pairwise Bonferroni-corrected comparisons showed that whereas the differences between unrelated and related words were not significant at the midline (*p* = 0.285), unrelated words elicited a significantly more positive-going wave than the one elicited by related words at both the left (*p* = 0.006) and right hemispheres (*p* = 0.007). In addition, the interaction effect between relatedness and latitude was significant (F (2, 50) = 8.261, *p* = 0.005, η_p_^2^ = 0.248). Pairwise Bonferroni-corrected comparisons showed that whereas there were no differences between unrelated and related words at the frontal region (*p* = 0.422), such differences were significant at both the central (*p* = 0.016) and posterior regions (*p* = 0.003). The three-way interaction effect among relatedness, hemisphere, and latitude also reached statistical significance (F (4, 100) = 9.364, *p* < 0.001, η_p_^2^ = 0.273). Further analysis showed that the differences between unrelated and related words were significant at the central (*p* = 0.002) and posterior regions (*p* = 0.006) and marginally significant at the frontal region (*p* = 0.054) of the left hemisphere. Regarding the midline, the differences were significant at the posterior region (*p* = 0.019) and not significant at the frontal and central regions (all *p*-values > 0.240). With respect to the right hemisphere, the differences were significant at the central (*p* = 0.002) and posterior (*p* < 0.001) regions, and not significant at the frontal region (*p* = 0.282). Finally, there was a significant three-way interaction effect among relatedness, type of relationship, and hemisphere, F (2, 50) = 5.811, *p* = 0.008, η_p_^2^ = 0.189. Further analyses showed that the differences between unrelated and related words in the semantic condition were not significant at either of the three levels of the hemisphere factor (all *p*-values > 0.116). In contrast, the differences between unrelated and related words in the form condition were significant at the right (*p* < 0.001) and left hemispheres (*p* < 0.001) as well as at the midline (*p* = 0.003). All other interaction effects were not significant (all *p*-values > 0.710).

### 2.3. Discussion

This experiment aimed to examine the extent to which highly proficient bilinguals of Spanish and Catalan activate the Spanish translation equivalents when they are presented with Catalan words. The results were clear-cut. Behavioral measures showed significant interference effects, both in RT and errors. These effects were larger in the semantic condition than in the form-related condition. ERP recordings, in turn, showed an effect of the semantic manipulation at the N400 component (unrelated words elicited a more negative-going wave than semantically related words) and an effect of the form manipulation at the LPC component (unrelated words elicited a more positive-going wave than form-related words).

This experiment was almost a replication of the experiment conducted by [11]. The main difference was that Moldovan et al. kept the first (Catalan) word constant and varied the second (Spanish) word across conditions, while the opposite strategy was used here. As explained above, this was the only way to use exactly the same materials in the two tasks included in this study. Another difference was that [11] included two semantically related conditions, which differed in the degree of semantic relationship between the two words in the pair, while only the more related condition was tested here. Our results agree with those of [11] both at a behavioral and at a neural level. The only difference concerns behavioral results. Concretely, the semantic interference effect was larger in this experiment than the form interference effect, while this was not the case in [11]. It should be noted, however, that the magnitude of the effects was very similar in both studies. Therefore, the present results indicate that the conceptual system has been activated. Indeed, considering that there is not any formal relationship between *cavall* (horse) and *burro* (donkey), the influence of the former in the processing of the latter needs to be conceptually mediated. Similar to the results of previous studies [7,8,9,10,11,12], these results support the RHM [1], indicating that highly proficient bilinguals directly access the conceptual system during translation recognition.

Regarding the form manipulation, which was the main focus of interest here, the behavioral results showed a clear interference effect, which indicated that the Spanish translation equivalent of the Catalan word had been activated. Indeed, the interference effect produced by the Catalan word “*fang”* can only be explained by the form similarity between its Spanish translation (*barro*) and the target Spanish word (*burro*), considering that there was neither any similarity in meaning between *fang* (mud) and *burro* (donkey) nor any similarity in form between the two words of the pair (*fang* and *burro*). Importantly, similar to in [9,11], the effects of the activation of the translation equivalent were observed in a late component (LPC), but not in the component that indicated semantic integration (N400). Therefore, this activation seems to occur after meaning has been accessed, suggesting that highly proficient bilinguals do not rely on the lexical route to perform a translation recognition task. In order to know if the activation of the translation equivalent is a by-product of the task employed or, rather, it is a general phenomenon, the same materials should be tested in a different task. This is what we did in Experiment 2.

## 3. Experiment 2. Lexical Decision Task

### 3.1. Materials and Methods

#### 3.1.1. Participants

Twenty-five participants (17 females, mean age = 22.1, SD = 2.8) took part in the experiment. They were undergraduate students at the Universitat Rovira i Virgili (Tarragona, Spain) who were paid to participate in the experiment. All of the participants signed an informed consent form before starting the experiment, and all participants had normal or corrected-to-normal vision and were right-handed. All participants were bilinguals of Spanish and Catalan. They completed the same questionnaire that was used in Experiment 1 to assess their proficiency in Catalan and Spanish, as well as the age of exposure to each language, and their language preference and use. The results of the questionnaire are represented in Table 4. All the participants were exposed to Catalan from birth (M = 0.60, SD = 1.04) and their average age of initial exposition to Spanish was 1.12 years (SD = 1.45). Ability ratings showed that they were all highly proficient in both languages (see Table 4). A paired comparison between Catalan (M = 6.72, SD = 0.51) and Spanish (M = 6.71, SD = 0.52) showed no differences between Catalan and Spanish in average reported proficiency (t (24) = 0.069, *p* > 0.940). The questionnaire also showed that both Catalan and Spanish were equally preferred (M = 4.05, SD = 0.73) and that participants used the two languages to the same extent (M = 4.04, SD = 0.87). Thus, participants were early balanced bilinguals of Spanish and Catalan, being highly proficient in both languages with no preference for one of the two languages. A series of independent samples *t*-tests showed that there were no differences between the participants of Experiments 1 and 2 in terms of average reported proficiency in the two languages, language preference, and language use (all *p*-values >0.810).

#### 3.1.2. Materials

The materials of the four experimental conditions (i.e., semantically related/unrelated, form related/unrelated) were the same as in Experiment 1. Due to the characteristics of the task (a lexical decision task), the NO trials of Experiment 1 (i.e., non-translation pairs) were YES trials in Experiment 2. This is because, in all of them, the second element of the pair (the target) was a word. The YES trials from Experiment 1 (i.e., translation pairs) were not used in Experiment 2. The NO trials in the present experiment were pairs of stimuli in which the first stimulus was a Catalan word and the second stimulus was a nonword. Thus, we created a set of 160 pronounceable and legal nonwords in Spanish from the 160 experimental targets (i.e., the second word of the pairs in the four experimental conditions), by using the Wuggy nonword generator [31]. Target words and nonwords were matched in length, number of syllables, subsyllabic structure, and transition frequencies.

#### 3.1.3. Procedure

The procedure, the software, and the presentation rates used in the present experiment were the same as that of Experiment 1. The only difference between the two experiments was the task that participants had to perform. In this case, it was a lexical decision task, where participants had to decide as rapidly and as accurately as possible whether or not the second visually presented letter string was a Spanish word. Participants were instructed to press the “yes” labeled key of a keyboard with the right hand if the second string of letters was a word, and to press the “no” labeled key of the keyboard with the left hand if it was not a word. The experiment began with a practice block consisting of 12 trials (6 word trials and 6 nonword trials). There were brief breaks after every 80 pairs. Participants were evenly distributed across the four counterbalanced lists.

#### 3.1.4. EEG Recording and Analysis

The same EEG equipment and parameters as in Experiment 1 were used in Experiment 2.

After artifact rejection, the percentage of trials retained for analyses was 87.50%, with 88.50% for the semantically related condition, 87.40% for the semantic control condition, 86.80% for the form related condition, and 87.30% for the form control condition. There was no significant effect of type of relationship or relatedness, nor were there any interaction effects between these factors (all *p*-values >0.31) on the percentage of trials retained for analysis.

### 3.2. Results

#### 3.2.1. Behavioral Results

RTs shorter than 200 ms and longer than 2000 ms were rejected. Further, scores that were 2.5 standard deviations above or below the mean value of each participant and each item were excluded. Only correct responses were included in the RT analyses. Mean reaction times (RTs) and error rates across the four critical conditions are shown in Table 5.

Separate 2 (type of relationship, semantic vs. form) * 2 (relatedness, related vs. unrelated) by-participant (F1) and by-item (F2) ANOVAs were performed for RTs and error rates. For the by-participant analyses, relatedness and type of relationship were within-participant factors. For the by-item analyses, type of relationship and relatedness were within-item factors.

##### Reaction Times

The results showed a significant effect of the type of relationship (F_1_(1, 24) = 22.182, *p* < 0.001, η_p_^2^ = 0.480, F_2_(1, 159) = 26.542, *p* < 0.001, η_p_^2^ = 0.143), indicating that responses to word pairs in the semantic condition (M = 705 ms, SE = 22.41) were slower than those in the form condition (M = 679 ms, SE = 21.54). The main effect of relatedness was also significant (F_1_(1, 24) = 10.595, *p* = 0.003, η_p_^2^ = 0.306, F_2_(1, 159) = 5.500, *p* = 0.020, η_p_^2^ = 0.033), suggesting that it took longer for participants to respond to unrelated (M = 698 ms, SE = 21.99) than to related words (M = 686 ms, SE = 21.77). The interaction effect between type of relationship and relatedness was also significant (F_1_(1, 24) = 8.389, *p* = 0.008, η_p_^2^ = 0.259, F_2_(1, 159) = 20.994, *p* < 0.001, η_p_^2^ = 0.117). Pairwise Bonferroni-corrected comparisons showed that there was a significant facilitation for the semantic condition (M = 28 ms, SE = 7.92, *p* = 0.001) but not for the form condition (M = 5 ms, SE = 5.51, *p* > 0.355).

##### Error Rates

The results showed a significant effect of the type of relationship (F_1_(1, 24) = 8.897, *p* = 0.006, η_p_^2^ = 0.270, F_2_(1, 159) = 6.770, *p* = 0.010, η_p_^2^ = 0.041), indicating that error rates for word pairs in the form condition (M = 5.89%, SE = 0.82) were higher than those in the semantic condition (M = 3.99%, SE = 0.69). The main effect of relatedness was marginally significant by participant (F_1_(1, 24) = 3.607, *p* = 0.070, η_p_^2^ = 0.131) and significant by item, (F_2_(1, 159) = 4.321, *p* = 0.039, η_p_^2^ = 0.026), suggesting that participants committed more errors in the unrelated condition (M = 5.59%, SE = 0.79) than in the related condition (M = 4.30%, SE = 0.74). The interaction effect between the type of relationship and relatedness was marginally significant by participant (F_1_(1, 24) = 3.264, *p* = 0.083, η_p_^2^ = 0.120) and significant by item (F_2_(1, 159) = 4.020, *p* = 0.047, η_p_^2^ = 0.025). Pairwise Bonferroni-corrected comparisons showed that the difference between the related and the unrelated words was significant in the semantic condition (M = 2.64, SE = 0.73, *p* = 0.002) but not in the form condition (M = 0.68, SE = 1.22, *p* > 0.900).

#### 3.2.2. ERP Results

As in Experiment 1, we conducted a four-way ANOVA with relatedness (related vs. unrelated), type of relationship (semantic vs form), hemisphere (left vs. midline vs. right), and latitude (frontal, central and parietal) as within-participant factors. As in the first experiment, only the main effect of the relatedness factor and significant interaction effects between this factor and the other three factors (i.e., type of relationship, hemisphere, and latitude) are reported. The main effect of the type of relationship and its interaction with the two topographical factors, as well as the main effects of interaction effects exclusively between topographical factors are not of primary relevance to the current work, and thus are not reported.

**Figure 4:**
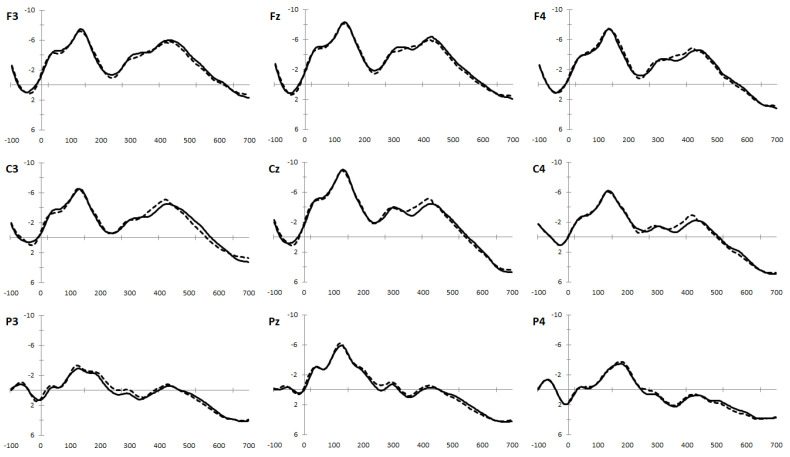
Grand average ERP waveforms for form-unrelated (dotted lines) and form-related words (solid lines) in Experiment 2. ERPs are plotted for nine equidistant representative electrodes within each region of interest. Negative voltage is plotted up. Onset of the Spanish word (i.e., the target) is indicated by the vertical calibration bar. Data are plotted from 100 ms prior to and 700 post stimulus onset.

**Figure 5:**
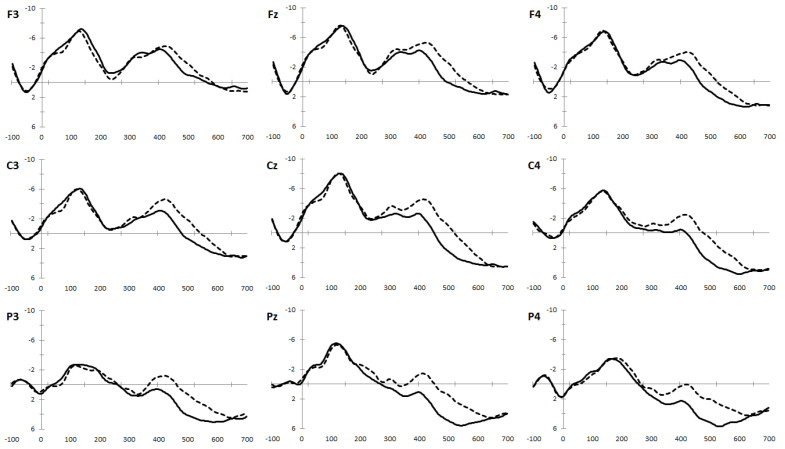
Grand average ERP waveforms for semantically unrelated (dotted lines) and semantically related words (solid lines) in Experiment 2. ERPs are plotted for nine equidistant representative electrodes within each region of interest. Negative voltage is plotted up. Onset of the Spanish word (i.e., the target) is indicated by the vertical calibration bar. Data are plotted from 100 ms prior to and 700 post stimulus onset.

**Figure 6:**
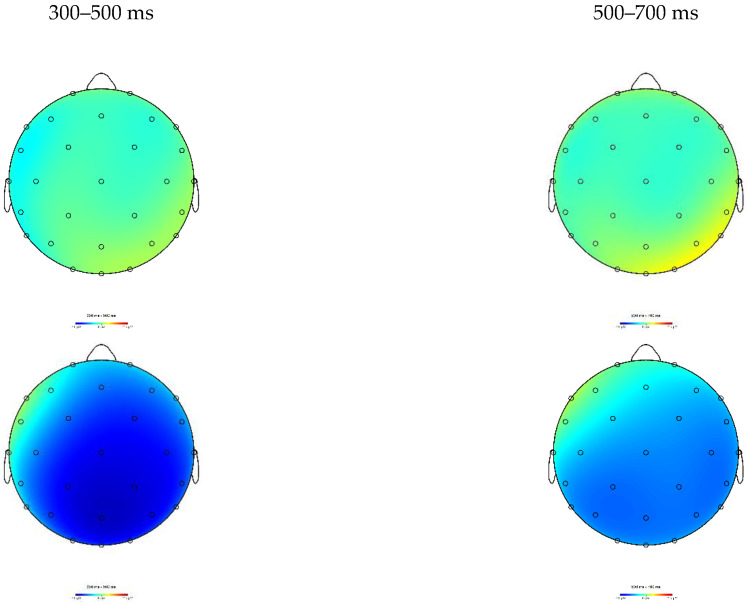
Scalp maps of relatedness effects (voltage differences were computed by subtracting the related from the unrelated condition) averaged across the N400 and the LPC time windows in Experiment 2 (upper row, the form condition and lower row, the semantic condition). Scale from −2.5 μV (blue) to 2.5 μV (red).

Grand-average ERP waveforms in a selection of electrodes are shown for the form condition (Figure 4) and the semantic condition (Figure 5). Figure 6 shows the scalp topographies of the mean amplitudes in the time windows for the N400 and the LPC components for the form condition (upper row) and the semantic condition (lower row).

Visual inspection of the ERP waveforms for the form condition (Figure 4) reveals that there are no differences between the waves elicited by unrelated words and the waves elicited by related words. In contrast, visual inspection of the ERP waveforms for the semantic condition (Figure 5) reveals that unrelated words as compared to related words elicit a clear modulation of the N400 component, starting at around 300 ms post stimulus. The topographic plots in Figure 6 show that there are no differences at the 300–500 ms time window between unrelated and related words in the form condition, whereas for the semantic condition unrelated words elicit a widely distributed negativity. Moreover, Figure 6 shows that, whereas for the form condition there are no differences between unrelated and related words, for the semantic condition, the negativity observed in the N400 time window remains visible, although less pronounced.

##### N400

The main effect of relatedness was significant (F (1, 24) = 4.254, *p* = 0.050, η^2^_p_ = 0.151). Unrelated words (M = −0.624 μV, SE = 0.62) elicited a more negative-going wave than related words (M = −0.098 μV, SE = 0.54). The interaction effect between relatedness and type of relationship was significant (F (1, 24) = 13.668, *p* = 0.001, η^2^_p_ = 0.363). Post hoc pairwise Bonferroni-corrected comparisons showed that, whereas unrelated words elicited a significantly more negative-going wave than related words in the semantic condition (*p* < 0.001), the differences between unrelated and related words were not significant in the form condition (*p* = 0.338). The ANOVA revealed a significant interaction effect between relatedness and latitude (F (2, 48) = 4.424, *p* = 0.038, η^2^_p_ = 0.156). Post hoc pairwise Bonferroni-corrected comparisons showed that, whereas the difference between unrelated and related words was not significant at the frontal region (M = −0.076 μV, SE = 0.34, *p* = 0.826), it was significant at both the central region (M = −0.605 μV, SE = 0.27, *p* = 0.035) and the posterior region (M = −0.896 μV, SE = 0.28, *p* = 0.005). The interaction effect among relatedness, type of relationship, and hemisphere was also significant (F (2, 48) = 3.887, *p* = 0.040, η^2^_p_ = 0.139). Pairwise Bonferroni-corrected comparisons revealed that the differences between the unrelated and the related words in the semantic condition were significant at the three levels of the factor hemisphere, being larger at the midline (M = −1.675 μV, SE = 0.46, *p* = 0.001) and the right hemisphere (M = −1.581 μV, SE = 0.35, *p* < 0.001), than at the left hemisphere (M = −0.875 μV, SE = 0.34, *p* = 0.017). In contrast, the differences between related and unrelated words in the form condition were not significant at either of the three levels of the hemisphere factor (all *p*-values > 0.163). All other interaction effects were not significant (all *p*-values > 0.107).

##### LPC

The ANOVA revealed that the main effect of relatedness was not significant (F < 1). No interaction effects between the relatedness factor and the other three factors reached statistical significance (all *p*-values > 0.206).

### 3.3. Discussion

This experiment aimed to examine whether the activation of the translation equivalent in highly proficient bilinguals was a by-product of the task (i.e., translation recognition) or rather, was a general phenomenon. The results showed a clear semantic facilitative effect, both at a behavioral and at a neural level, and a total absence of any form-related effect.

The semantic facilitative effect was evidenced in this experiment by shorter RTs and higher accuracy in the related condition as compared with the unrelated condition, as well as by a reduced amplitude of the N400 component in the former as compared to the latter. These results agree with previous findings that have shown that the presentation of words in one language facilitated the processing of semantically related words in the other language [7,14,16,17,18]. Considering that there was not any formal relationship between the Catalan word and the Spanish word in each pair, the results of this experiment support the RHM [1], indicating that highly proficient bilinguals directly access the conceptual system. They also support another influential model, the distributed representational model (DRM, [32]), which proposes a shared and distributed conceptual system between the two languages of a bilingual speaker.

In contrast with the semantic manipulation, in this experiment, there were no effects at all of the formal manipulations (either at a behavioral level or at a neural level). These results suggest that the activation of the translation equivalents observed in Experiment 1 might be due to the characteristics of the task (a task involving the comparison of two words to decide about their translation equivalents status), and not a general phenomenon in highly proficient bilinguals.

## 4. General Discussion and Conclusions

This study investigated the extent to which highly proficient bilinguals of Spanish and Catalan activate the Spanish translation equivalents when they are presented with Catalan words, regardless of the task at hand. Participants performed a translation recognition task (Experiment 1) or a primed lexical decision task (Experiment 2) where the relationship between the first presented (Catalan) word and the second presented (Spanish) word was manipulated. Semantic relatedness produced an interference effect in the translation recognition task and a facilitation effect in the primed lexical decision task. These effects were observed both at a behavioral (RT and errors) and at a neural level (modulation of the N400 component). Form relatedness produced an interference effect only in the translation recognition task, which was indicated by RT and error rates, as well as by a modulation of the LPC component. In contrast, there were no effects of the formal manipulation in the primed lexical decision task.

The semantic effects obtained in this study are in line with previous research that has shown that semantic relatedness between words across languages produces an interference effect in translation recognition tasks [7,8,9,10,11,12]. That is, participants were slower and less accurate in rejecting semantically related words as translation equivalents than unrelated words. Our findings are also in line with previous studies that have shown a semantic facilitative effect in primed lexical decision tasks [14,15,16,17,18]. That is, participants decided faster that a particular string of letters was an actual Spanish word when it was preceded by a semantically related Catalan word. These effects are both indicative that highly proficient bilinguals directly access the conceptual system [1] and that there is a shared conceptual system between the two languages [32]. According to the DRM, meaning is represented as a set of distributed features. The presentation of a Catalan word (e.g., *cavall*, horse) activates all its meaning features. Hence, the features in common with other semantically related words (e.g., *burro*, donkey) are preactivated. This may produce facilitation or interference effects, depending on the nature of the task. In a primed lexical decision task, preactivation of features facilitates lexical access and the recognition of the target word. This semantic priming effect has been repeatedly observed both in the native language (e.g., [15,33]) as well as across languages [14,15]. Furthermore, it is sensitive to the degree of semantic relatedness between two words [14,33]. In contrast, in the translation recognition task, this preactivation may lead participants to wrongly decide that the semantically related Spanish word is the correct translation of the Catalan word, or to need more time to reject that word as the correct translation. This is because, unlike the lexical decision task, the translation recognition task requires a fine semantic distinction between words to be performed correctly. Interestingly, despite the opposite behavioral patterns, the effects in the N400 component had the same direction in both experiments: Semantically related words across languages elicited a smaller N400 relative to unrelated controls, indicating that it is easier to integrate the meaning of a semantically related word than that of an unrelated word. Again, this effect agrees with previous research with the semantic priming paradigm (e.g., [34]) and the translation recognition task [9,11], where a modulation of the N400 effect by the degree of semantic similarity has also been reported (i.e., the effects are larger as the degree of semantic relatedness becomes higher [11].

The performance of these highly proficient bilinguals when translating Catalan words was affected by the semantic manipulation and also by the form manipulation, in line with previous findings [7,8,9,10,11,12]. Importantly, similar to the results in [11], the form effects appeared in a late ERP component, indicating that this activation occurred after meaning had been accessed. Therefore, these results would not challenge the RHM, because the activation of the Spanish translation equivalents was not necessary to access meaning. The most innovative result of this study is that such activation was not observed in a primed lexical decision task. Indeed, if the Spanish translation of *fang* (*barro*) was activated when *fang* was presented, it should have had an effect in the processing of *burro* (i.e., in the decision about the lexical status word or nonword of *burro*). This is a common finding in studies where the orthographic similarity between primes and targets is manipulated in a priming paradigm (e.g., [19,20,21,22,23]). The lack of such an effect either at a behavioral or at a neural level suggests that this activation may be task dependent, and a logical consequence of the characteristics of the translation recognition task. Indeed, to decide whether the two words in a pair are translation equivalents, the Spanish word needs to be compared to the Spanish translation of the Catalan word presented immediately before. This comparison is not needed in a primed lexical decision task where participants make decisions about the second word, without any reference to the first word or their translation equivalent.

To conclude, highly proficient bilinguals of Catalan and Spanish seem to activate the translation equivalent after accessing meaning when they perform a translation recognition task. This activation appears to be a by-product of the requirements of the task because it is not observed in a primed lexical decision task. The activation of the translation equivalent after meaning access suggests that these bilinguals do not rely on the lexical route to perform the translation recognition task, indicating that form-related effects in highly proficient bilinguals [7,8,9,10,11] do not challenge the RHM. A limitation of this study is that only two tasks were included and that only a translation direction was assessed. Future studies should examine the performance of bilingual populations differing in their proficiency on various tasks and in the two directions (from L1 to L2 and the other way around) to assess the generalizability of these findings.

## Figures and Tables

**Table 1:** Mean and standard deviation (in parentheses) of participants’ self-rated proficiency in Catalan and Spanish.

Skills	Catalan	Spanish
Listening	6.88 (0.43).	6.85 (0.54)
Speaking	6.62 (0.89)	6.65 (0.84)
Reading	6.85 (0.47)	6.81 (0.57)
Writing	6.58 (0.90)	6.65 (0.56)
Average	6.73 (0.58)	6.74 (0.57)

Note: The anchor points for the 7-point Likert scale were 1 = ”very poor” and 7 = ”native-like”.

**Table 2:** Means and standard deviation (in parentheses) for stimuli properties in the critical conditions.

Condition	Related		Unrelated	
	Length	Frequency	Length	Frequency
Semantic	6.5 (1.9)	34.1 (89.4)	6.4 (1.9)	34.3 (99.6)
Form	5.6 (1.9)	54.7 (132.4)	5.6 (1.9)	40.6 (108.1)

Note: Word frequency is based on the frequency norms of [28] and retrieved from NIM [25]. Length is measured as the number of characters in a word.

**Table 3:** Mean reaction times (ms) and error rates (%) in the four experimental conditions (standard deviations in parentheses).

Condition	RTs	Error Rates
Semantic		
Related	763 (159)	25.5 (10.0)
Unrelated	673 (124)	1.3 (2.1)
Form		
Related	715 (112)	6.6 (5.2)
Unrelated	649 (115)	0.4 (1.1)

**Table 4:** Mean and standard deviation (in parentheses) of participants’ self-rated proficiency in Catalan and Spanish.

Skills	Catalan	Spanish
Listening	6.84 (0.37)	6.80 (0.50)
Speaking	6.72 (0.54)	6.60 (0.76)
Reading	6.88 (0.33)	6.80 (0.50)
Writing	6.44 (1.12)	6.64 (0.57)
Average	6.72 (0.59)	6.71 (0.58)

Note: The anchor points for the 7-point Likert scale were 1 = ”very poor” and 7 = ”native-like”.

**Table 5:** Mean reaction times (ms) and error rates (%) in the four experimental conditions (standard deviations in parentheses).

Condition	RTs	Error Rates
Semantic		
Related	665 (104)	2.7 (3.2)
Unrelated	693 (114)	5.3 (4.5)
Form		
Related	708 (117)	5.9 (5.2)
Unrelated	703 (108)	5.9 (5.0)

## Data Availability

The complete list of experimental materials and the behavioral and ERP results of Experiments 1 and 2 are available at: https://osf.io/rvx48/.
